# M1 intestinal macrophages-derived exosomes promote colitis progression and mucosal barrier injury

**DOI:** 10.18632/aging.205672

**Published:** 2024-03-26

**Authors:** Rui Du, Sihan Chen, Chenyang Han, Zhongmei He, Hongyan Pei, Yang Yang

**Affiliations:** 1 College of Chinese Medicinal Materials, Jilin Agricultural University, Changchun 130118, China; 2 Department of Pharmacy, The Second Affiliated Hospital of Jiaxing University, Jiaxing 314001, China; 3 Department of Cardiology, Shenyang Medical College Affiliated Second Hospital, Shenyang 314005, China

**Keywords:** M1 macrophages, exosomes, inflammation, colitis

## Abstract

Aim: This work aimed to investigate the role of M1 intestinal macrophages-derived exosomes (M1-Exo) in colitis and its mechanism.

Methods: M1 polarization of intestinal macrophages was induced *in vitro*, and their exosomes were extracted and identified. Thereafter, the DSS-induced colitis mouse model was built. Each mouse was given intraperitoneal injection of exosomes, and then mouse weight and DAI were dynamically monitored. In addition, the levels of cytokines were detected by ELISA. After treatment with the TLR4 inhibitor Resatorvid, the effects of M1 macrophages-derived exosomes were observed. Besides, the mouse intestinal epithelial cells were cultured *in vitro* for observing function of M1-Exo.

Results: M1-exo aggravated the colitis and tissue inflammation in mice, activated the TLR4 signal, and destroyed the mucosal barrier. But M0 macrophages-derived exosomes (M0-Exo) did not have the above effects. Resatorvid treatment antagonized the roles of M1-exo. Moreover, as confirmed by cellular experiments *in vitro*, M1-exo destroyed mucosal barrier.

Conclusion: M1-exo serve as the pro-inflammatory mediator, which can promote mouse colitis progression by activating TLR4 signal.

## INTRODUCTION

Inflammatory bowel disease (IBD) represents the idiopathic, chronic and relapsed intestinal inflammatory disorder involving ileum, rectum and colon. IBD consists of Crohn’s disease as well as ulcerative colitis [1, 2]. Mucosal barrier dysfunction has been currently recognized as a major factor leading to IBD [3]; to be specific, antigens stimulate the intestinal mucosal injury, enhance the mucosal permeability, while stimulating various inflammatory factor production [4], thus promoting involvement of the body in the adaptive immune response network.

Macrophages are an important immune cells, which are divided into M1 or M2 subtype in line with their different activation modes. M1 macrophages are activated via interferon-γ (IFN-γ) and tumor necrosis factor (TNF)-α to secrete interleukin [5], and can promote inflammatory response as the effector cells [6]. In the IBD state, macrophages can recognize pathogenic microorganisms and release inflammatory factors IL-1α, IL-1β and TNF-α to increase inflammatory response. Consequently, M1 macrophages have critical effect on promoting inflammation during IBD, and they are also the major cell type resulting in IBD progression [7]. Exosome is a medium for inter-cellular signal connection. At present, exosome-induced diseases have been extensively investigated [8], however, apart from the release of excessive amounts of inflammatory factors, the role of exosomes in IBD remains unknown. Consequently, the present work focused on revealing function of M1 intestinal macrophages-derived exosomes (M1-Exo) in IBD progression and their associated mechanism.

## MATERIALS AND METHODS

### M1 cell induction and exosome extraction and identification

Intestinal macrophages (Procell Biotechnology Co., Ltd., Wuhan, China) were cultivated within DMEM complete medium that contained 10% FBS and incubated within a tri-gas incubator at 37°C with 5% CO_2_ till adherence. Following cell passage, macrophages were transferred into the culture flask, the complete medium was replaced after achieving cell adherence. During macrophage M1 polarization, we induced cells using 100 ng/ml LPS (Sigma, USA) along with 30 ng/ml recombinant IFN-γ protein (KALANG, USA), then observed cell morphology, and collected both cell culture and cells 72 h later. Thereafter, cells were further cultured in fresh medium for another 24 h. While M1 and M2 cell marker expression was measured through ELISA.

After the successful induction of M1 cells, cells were further cultured with DMEM complete medium without exosome serum for 48 h in the culture flask. After collection of cells and cell medium, exosomes were isolated through ultra-high speed centrifugation in line with SBI kit instructions. The M1 cells-derived exosomes were defined as M1-Exo, meanwhile, exosomes were also extracted from the non-induced M0 cells by the above method, which were defined as M0-Exo.

### Mouse model

The wild-type C57BL/6 female mice (21 ± 4 g) were selected in modeling. Following 1-week adaptive feeding, animals were classified as Control, DSS, M0-Exo or M1-Exo group. M0-Exo and M1-Exo groups were injected with 10 mg/kg total protein via the intraperitoneal route once every other day for 13 times altogether. To induce colitis by DSS, mice were given 2.5% DSS to construct a mouse model of chronic colitis. Specifically, the animals were allowed to drink 2.5% DSS for five days in one cycle, and purified water in rest days. In total, there were four cycles, and on days one, three, and five of each cycle, DAI scores and body weights of mice were under dynamic measurements. Mice were sacrificed on day 29 through decapitation for subsequent experiments. Intestinal tissue was separated between distal ileum and rectum, and rinsed with PBS (Sigma, USA) solution thrice for later detection.

During mechanism research, animals were classified as Control, DSS, M1-Exo, or Res+M1-Exo group. Of them, Resatorvid (Res) was the TLR4 signal inhibitor, which was given simultaneously with M1-Exo through intragastric administration. The above operations were applied in the remaining treatments.

### Determination of body weight, DAI pathological score and intestinal length of mice

On days one, three and five of each cycle, body weights were under dynamic measurement, then the weight to initial weight ratio (%) was determined. Mouse pathological status was rated by DAI score, which was determined on days one, three and five in every cycle in line with DAI scoring standards. For colon length measurement, colon tissue was collected after mouse sacrifice to determine the length, and result was represented by cm. For colon morphological score, damage morphological score of separated colon was rated in line with specific standards.

### Hematoxylin and eosin (H&E) staining

Colon tissue was collected when mice were sacrificed, which were processed by paraffin embedding and preparation in 4-μm consecutive sections for subsequent use. Later, the sections were subjected to xylene deparaffinization, gradient ethanol dehydration (100%, 95% and 80% successively), 2-min washing by running water, and 3-min staining using hematoxylin. Sections were later rinsed by running water for a 2-min period, followed by 2-s treatment using 1% hydrochloric alcohol, 2-min washing by running water, 20-s treatment using 1% ammonium hydroxide, 10-s staining with 0.5% eosin alcohol, gradient alcohol dehydration, xylene transparentizing and neutral gum mounting. At last, pathological change in intestinal tissue was monitored with an optical microscope.

### FITC-D

After feeding, fluorescein isothiocyanate-dextran (FITC-D, MW 4000) was administrated through gavage in mice, then FITC-D concentration was detected to analyze change of intestinal mucosal permeability. Following intervention, food and water fasting was conducted 4 h before mouse sacrifice. After gavage of FITC-D, serum was harvested from each mouse, fluorescence density in every sample was measured by the fluorescence spectrophotometer, while FITC-D concentration in the serum was detected.

### Enzyme linked immunosorbent assay (ELISA)

The intestinal tissue was washed with PBS twice, and grounded with liquid nitrogen till no granule was observed, followed by 30-min lysis using 1 ml NP-40 cell lysis buffer (Beyotime Biotechnology Co., Ltd., Shanghai, China) on ice to collect supernatant protein liquid. Finally, ELISA kits (Nanjing Jiancheng Institute of Biology, Nanjing, China) were employed to determine cytokine levels following instructions.

### Western-blot

Protein content was quantified, then protein separation was completed through electrophoresis, followed by transfer on PVDF membranes. Afterwards, PVDF membranes were subjected to 2-h blocking using 5% defatted milk powder, overnight incubation using TBST-diluted primary antibodies (dilution, 1:800, v/v) under 4°C, and later incubation using TBST-diluted, HRP-labeled IgG secondary antibody (dilution, 1:2000, v/v; Abcam, Massachusetts, USA). Afterwards, the chemiluminescent immunoassay was conducted for protein blot detection, Image Pro-Plus 6.0 software was employed to analyze optical density, and GAPDH was used as internal control. Data were represented by optical density ratio of target protein to internal control protein.

### Statistical analysis

Measurement data were represented by (± s) and tested by SPSS 17.0 statistical software. Following homogeneity test of variances, two independent sample t-test and one-way ANOVA were employed to compare two and multiple groups, separately. Then, LSD was employed for subsequent pairwise comparison. *P* < 0.05 (two-sided) represented significant difference.

### Availability of data and materials

The data that support the findings of this study are available from the corresponding author upon reasonable request.

## RESULTS

### Intestinal macrophage polarization and characterization results

**Figure 1:**
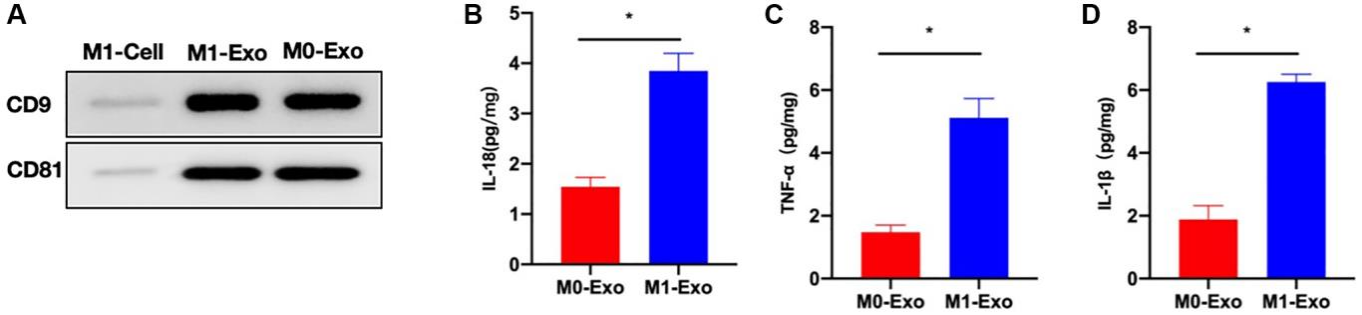
**M1 polarization of intestinal macrophages and characterization of exosomes.** (**A**) CD9, CD81 were highly expressed in exosomes. (**B**–**D**) ELISA (*n* = 3). Inflammatory cytokines IL-18, IL-1β, and TNF-α levels of M1-Exo group increased relative to M0-Exo group. ^*^P < 0.05 between two groups.

Marker detection results indicated that CD9, CD81 were highly expressed in exosomes (Figure 1A). As discovered after exosomal analysis, inflammatory cytokines (IL-18, IL-1β, and TNF-α) of M1-Exo group increased relative to M0-Exo group, and M1-Exo served as an inflammatory mediator (Figure 1B–1D).

### M1-Exo promoted colitis progression

**Figure 2:**
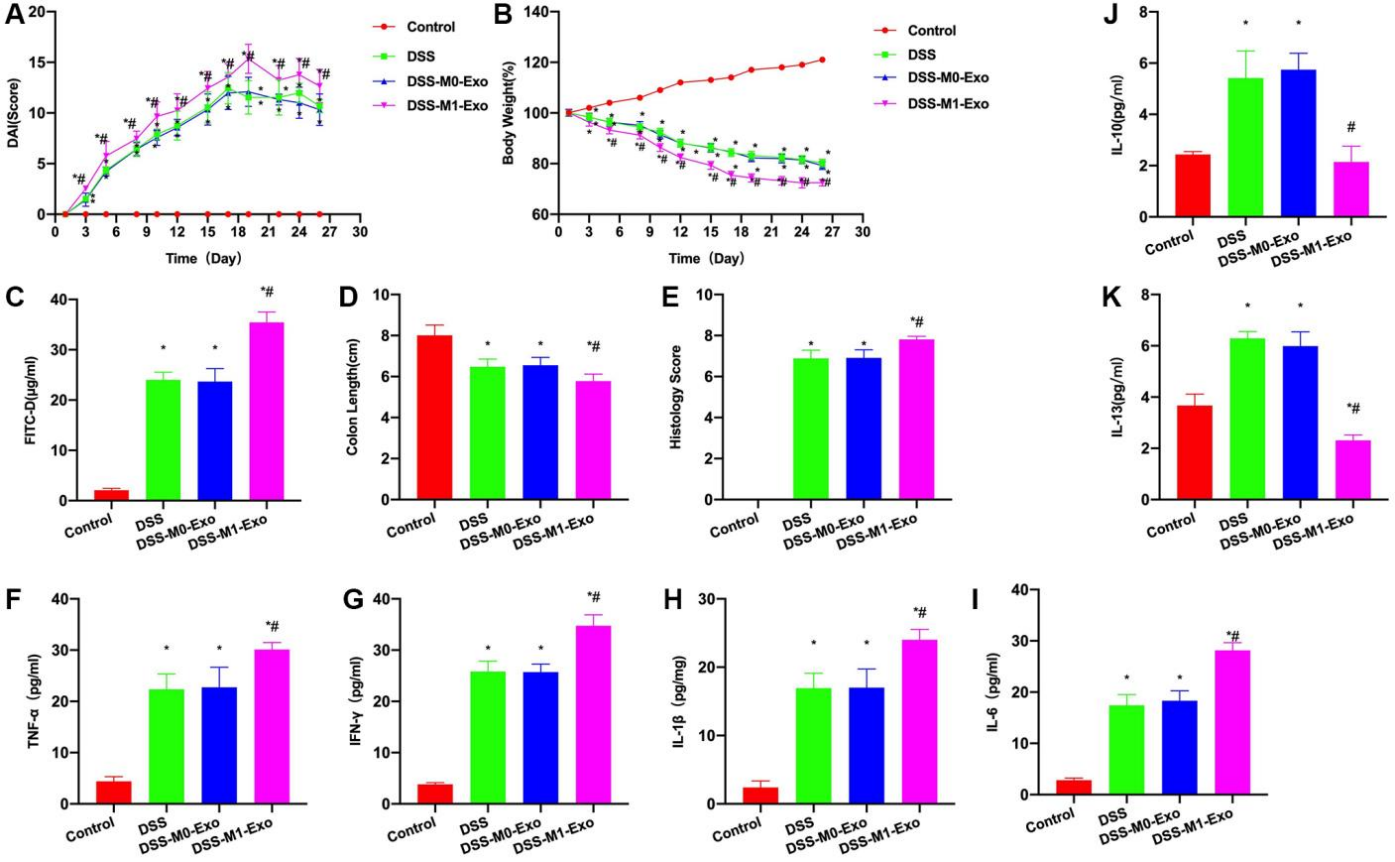
**M1-Exo promotes colitis progression.** (**A**) DAI score (*n* = 10). DAI score of DSS group increased, M0-Exo and DSS groups did not exhibit any significance, but DAI score of M1-Exo group apparently increased relative to DSS group. (**B**) Body weight measurement (*n* = 10). Body weight of DSS mice gradually declined, and M1-Exo group showed more weight loss than DSS group. (**C**) FITC-D (*n* = 10). FITC-D level of DSS group was up-regulated, but was lower than M1-Exo group. (**D**–**E**) Pathology and intestinal length (*n* = 10). Colon length of DSS group was apparently shortened, and that in M1-Exo group was shortened. Meanwhile, pathological score results also revealed the higher score in M1-Exo group than DSS group. (**F**–**K**) ELISA (*n* = 10). IFN-γ, IL-6, IL-1β and TNF-α levels of DSS group increased relative to Control group, which further increased in M1-Exo group compared with DSS group. IL-10 and IL-13 expression of DSS group increased but decreased in M1-Exo group. ^*^P < 0.05 versus Control group, ^#^*P* < 0.05 versus DSS group.

After intraperitoneal injection M0-Exo and M1-Exo in mice, results revealed that M0-Exo did not significantly promote colitis in M0-Exo group, while M1-Exo promoted colitis progression. According to DAI score analysis, the DAI score of DSS group increased with time, M0-Exo and DSS groups did not exhibit ant significant difference, but DAI score of M1-Exo group apparently increased relative to DSS group (Figure 2A). In body weight measurement, DSS group had gradually reduced body weight, while M0-Exo and DSS groups did not exhibit any significant difference, and M1-Exo group had more weight loss than DSS group (Figure 2B). Moreover, FITC-D detection found that the mucosal barrier permeability of DSS mice increased, and FITC-D level increased, while the FITC-D level in M1-Exo group increased relative to DSS group (Figure 2C). Colon length of DSS group was markedly shortened, that of M1-Exo group was shortened, and pathological score analysis revealed the higher score of M1-Exo group than DSS group (Figure 2D, 2E). Inflammatory factors were detected, as a result, IFN-γ, IL-6, IL-1β and TNF-α levels of DSS group increased IL-6, IL-1β and TNF-α levels of DSS group increased relative to Control group, and these levels of M1-Exo group further increased compared with DSS group (Figure 2F–2I). Although IL-10 and IL-13 levels of DSS group increased, they decreased in M1-Exo group (Figure 2J, 2K).

**Figure 3:**
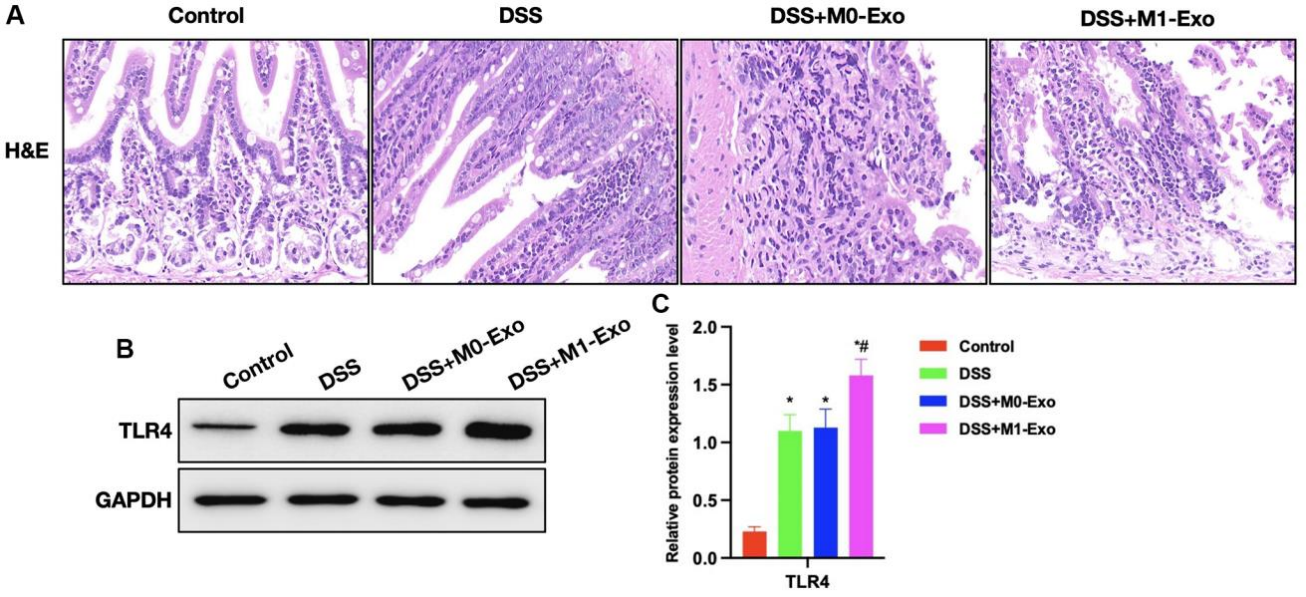
**Role of M1-Exo in the pathology of colitis.** (**A**) H&E (*n* = 5). Intestinal tissue in DSS group showed apparent edema, inflammatory tissue, and epithelial cell injury, while that in M1-Exo group showed aggravated mucosal destruction and obvious inflammatory response. (**B**, **C**) Relative protein levels (*n* = 5). TLR4 of DSS group were up-regulated, and further increased in M1-Exo group. ^*^P < 0.05 versus Control group, ^#^*P* < 0.05 versus DSS group.

According to H&E staining, intestinal tissue of DSS group showed apparent edema, inflammatory tissue, and epithelial cell injury, while that in M1-Exo group showed aggravated mucosal destruction and obvious inflammatory response (Figure 3A). In comparison, TLR4 expression was further up-regulated in M1-Exo group (Figure 3B, 3C).

### Suppressing TLR4 signal antagonized the M1-Exo promoted colitis progression

**Figure 4:**
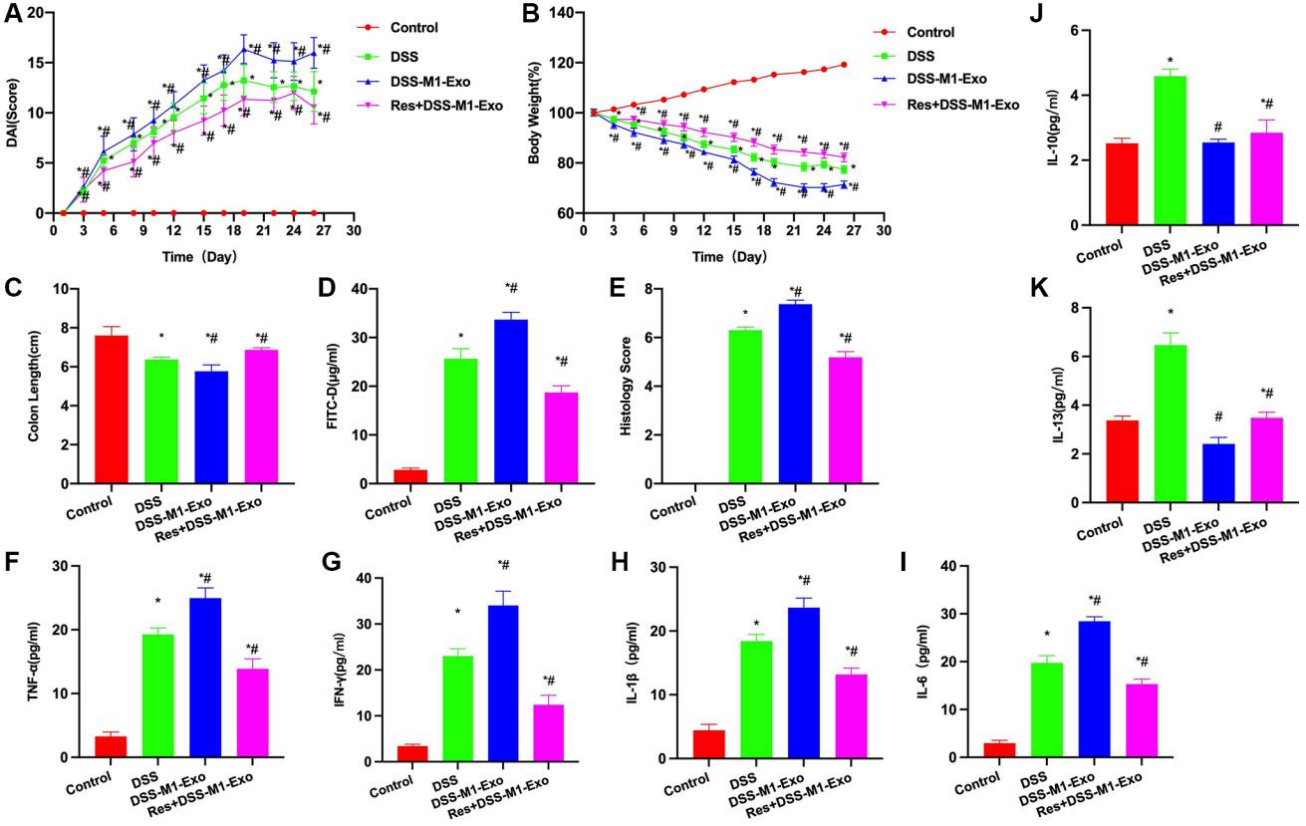
**Suppressing TLR4 signal antagonizes M1-Exo.** (**A**) DAI score (*n* = 10). DAI score of Res+M1-Exo group decreased relative to M1-Exo group. (**B**) Body weight measurement (*n* = 10). Weight loss of Res+M1-Exo group decreased relative to M1-Exo group. (**C**) Intestinal tissue length (*n* = 10). Res+M1-Exo group had apparently higher length than M1-Exo group. (**D**) FITC-D (*n* = 10). Res reduced mucosal barrier permeability, and FITC-D concentration of Res+M1-Exo group dramatically decreased relative to M1-Exo group. (**E**) Pathological score (*n* = 10). Res reduced the score, lower than that in M1-Exo group. (**F**–**K**) ELISA (*n* = 10). Res decreased inflammatory factor levels IFN-γ, IL-6, IL-1β and TNF-α, and elevated those of IL-10 and IL-13, with significant differences relative to M1-Exo group. ^*^P < 0.05 versus Control group, ^#^*P* < 0.05 versus DSS group.

Res was used to inhibit the TLR4 signal, it was found that inhibiting TLR4 signal antagonized M1-Exo, and improved mouse colitis. According to DAI score analysis, DAI score of Res+M1-Exo group decreased compared with M1-Exo group (Figure 4A). Body weight score results indicated that Res+M1-Exo group had lower weight loss than M1-Exo group (Figure 4B). Additionally, intestinal length detection revealed that, intestinal length in Res+M1-Exo group apparently increased relative to M1-Exo group (Figure 4C). FITC-D detection results suggested that, Res reduced the intestinal barrier permeability, and FITC-D level of Res+M1-Exo group dramatically decreased compared with M1-Exo group (Figure 4D). Pathological score analysis demonstrated that Res reduced the score, lower than in M1-Exo group (Figure 4E). Based on cytokine detection, Res decreased inflammatory factor contents, decreased IFN-γ, IL-6, IL-1β and TNF-α levels, and up-regulated IL-10 and IL-13 expression, with significant difference relative to M1-Exo group (Figure 4F–4K).

**Figure 5:**
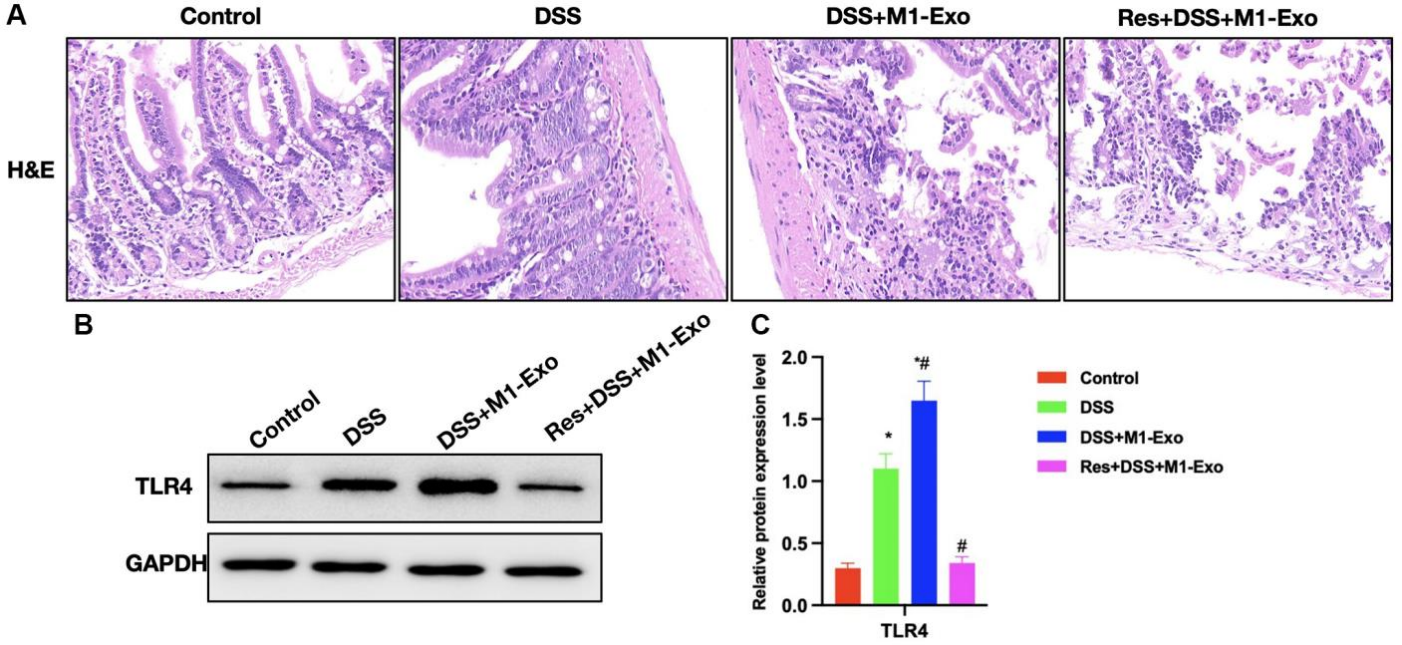
**Suppressing TLR4 signal antagonizes the effect of M1-Exo on tissue pathology.** (**A**) H&E (*n* = 5). Res improved mouse intestinal inflammation and epithelial cell injury, and decreased the inflammatory response in intestinal tissue. (**B**, **C**) Relative protein levels (*n* = 5). Res reduced TLR4 expression. ^*^P < 0.05 versus Control group, ^#^*P* < 0.05 versus DSS group.

As revealed by H&E staining, Res improved intestinal inflammation and epithelial cell injury in mice, accompanied by reduced inflammatory response in intestinal tissue (Figure 5A) Relative protein expression analysis indicated that Res down-regulated TLR4 (Figure 5B, 5C).

## DISCUSSION

Macrophages are an important immune cell type, which possess effects such as pathogen phagocytosis, antigen presentation and cytokine secretion, among which, M1 cells are considered as an important link in the colitis genesis and progression [9, 10]. Macrophages are polarized and differentiate into M1 and M2 cells by the surrounding T lymphocytes cofactors and pathogens, with M1 macrophages mainly exerting the pro-inflammatory effect [11]. LPS is the major activation signal of M1 macrophages, which can activate the dependent signals like MyD88 and Mal via TLR4 signal [12] to promote inflammatory factor production [13], mainly IFN-γ, IL-1β and IL-6 [14]. In colitis, M1 macrophages exist in a large amount in the lamina propria of colonic mucosa, compared with normal mucosa, these macrophages exhibit the higher activated state, which can generate potent pro-inflammatory effect [15]. Previous studies have discovered that MD2 can serve as the helper TLR4 protein to promote colitis progression [16, 17]. Existing studies only reveal the role of M1 macrophages in releasing inflammatory factors, but the function of exosomes has not been clearly reported. TLR4 signal can be activated by multiple inflammatory mediators, and it is also an important factors resulting in mucosal barrier injury in colitis [18]. The pro-inflammatory factors released in intestinal M1 macrophages during inflammation activate immune cells for suppressing invasion of microorganisms [19], and impact tight junction protein distribution, expression and composition, thus impacting the function of intestinal mucosal barrier [20]. Intestinal mucosal barrier is formed via the tight junction of single-layer columnar epithelial cells consisting of intestinal epithelial cells [21].

Exosomes, as the media of intercellular communication and substance delivery, play an important role in disease progression [22]. Our research discovered that the M1-Exo served as the inflammatory mediator to promote colitis progression. M1 and M0 cells-derived exosomes were isolated, and it was found that, M1-Exo expressed inflammatory cytokines like IL-18, IL-1β and TNF-α at high levels. This is because that excessive inflammatory factors are expressed in M1 polarization, while exosomes are the vesicae including excessive inflammatory cytokines, leading to up-regulation of these inflammatory cytokines within exosomes. In animal experiment, we discovered after injection of exosomes that, M0-Exo did not significantly affect DSS-induced mouse colitis, and DAI score, mouse body weight and pathological score were not significantly different from DSS group. But M1-Exo promoted colitis progression, and further aggravated tissue inflammation and mucosal barrier injury. Meanwhile, the FITC-D levels in mice increased, and the inflammatory factors were released at a large amount. IL-10 and IL-13 account for anti-inflammatory factors, which were up-regulated in DSS group, but their expression was suppressed in M1-Exo group. M1-Exo mainly exerted its effect through TLR4 signal. After treatment with TLR4 inhibitor, the effect of M1-Exo was significantly antagonized, mucosal barrier injury and tissue inflammation in mice were suppressed, and the mouse life state and DAI scores were improved. Therefore, it is suggested that M1-Exo exerts its effect through activating TLR4.

## CONCLUSION

In this study, it is discovered that M1 macrophages-derived exosomes can serve as an inflammatory mediator, which contains high levels of inflammatory factors. These exosomes activate TLR4 signal for promoting colitis progression and mucosal barrier injury. Suppressing M1 cells-derived exosome delivery can become a new thinking of colitis treatment.
